# Mechanism of N-Methyl-N-Nitroso-Urea-Induced Gastric Precancerous Lesions in Mice

**DOI:** 10.1155/2022/3780854

**Published:** 2022-03-16

**Authors:** Sheng-Xiong Zhang, Wen Tian, Yuan-Liang Liu, Jia-Hui Ni, Dan Zhang, Hua-Feng Pan, Zi-Ming Zhao, Bo Ai, Zhe-Sheng Chen, Li-Zhu Lin, Wei Liu

**Affiliations:** ^1^Guangdong Work Injury Rehabilitation Hospital, Guangzhou 510440, China; ^2^Lingnan Medical Research Center, Guangzhou University of Chinese Medicine, Guangzhou 510405, China; ^3^Guangdong Province Engineering Technology Research Institute of T.C.M, Guangzhou 510000, China; ^4^Tongji Medical College of Huazhong University of Science and Technology, Huazhong University of Science and Technology Tongji Medical College, Wuhan 430030, China; ^5^Institute for Biotechnology, St. John's University, Queens, New York 11439, USA; ^6^Integrative Cancer Centre, The First Affiliated Hospital of Guangzhou University of Chinese Medicine, Guangzhou 510095, China; ^7^Mathematical Engineering Academy of Chinese Medicine, Dongguan 523000, China

## Abstract

Early diagnosis and treatment of gastric precancerous lesions (GPL) are key factors for reducing the incidence and morbidity of gastric cancer. The study is aimed at examining GPL in mice induced by N-methyl-N-nitroso-urea (MNU) and to illustrate the underlying mechanisms of tumorigenesis. In this study, we utilized an in vivo MNU-induced GPL mouse model, and histopathological changes of the gastric mucosa were observed by hematoxylin and eosin (H&E-stain) and alcian blue (AB-PAS-stain). The level of miR-194-5p in the gastric mucosa was determined by real-time polymerase chain reaction. We used transmission electron microscopy to observe the effects of MNU on gastric chief cells and parietal cells. We performed immunohistochemical detection of HIF-1*α*, vWF, Ki-67, and P53, while the changes in the protein expression of key genes in LKB1-AMPK and AKT-FoxO3 signaling pathways were detected by western blot analysis. We demonstrated that the miR-194-5p expression was upregulated under hypoxia in GPL gastric tissues, and that a high miR-194-5p expression level closely related with tumorigenesis. Mechanistically, miR-194-5p exerted the acceleration of activities related to metabolic reprogramming through LKB1-AMPK and AKT-FoxO3 pathways. Furthermore, similar to miR-194-5p, high expression levels of AMPK and AKT were also related to the metabolic reprogramming of GPL. Moreover, we revealed the correlation between the expression levels of miR-194-5p, p-AMPK*α*, p-AKT, and FoxO3a. These findings suggest that miR-194-5p/FoxO3 pathway is important for the reversal of metabolic reprogramming in GPL. Thus, exploring strategies to regulate the miR-194-5p/FoxO3a pathway may provide an efficient strategy for the prevention and treatment of GPL.

## 1. Introduction

Gastric cancer is the third leading cause of cancer mortality worldwide in men and the fifth leading cause of cancer mortality in women [1]. Intestinal-type gastric cancer is preceded by GPL, including chronic atrophic gastritis (CAG), intestinal metaplasia (IM), and dysplasia (Dys), finally progressing to cancer [2]. Among patients who undergo gastroscopy with biopsy for clinical indications, 2% of patients with CAG, 2.56% of patients with IM, and 5.26% patients with Dys will develop cancer within 20 years [3]. Little is known about the mechanisms of gastric cancer tumorigenesis and development, and GPL could be an important subject for studying the pathogenesis of gastric cancer.

MicroRNAs bind to target genes and subsequently regulate the gene expression; as a result, they play a key role in a variety of cell developmental processes and in tumorigenesis. Previous studies have identified differentially expressed miRNAs during early gastric cancer and have demonstrated the specific expression of miR-194-5p in the gastrointestinal tract, which was induced during the process of intestinal epithelial cell differentiation [4, 5]. miR-194-5p plays an important role in the invasion and progression of gastric cancer [6]. However, the molecular mechanisms of miR-194-5p in the carcinogenesis of GPL remain to be elucidated.

Metabolism provides information about the progression and treatment of cancer. Under a hypoxic microenvironment, the forkhead transcription factors of class O3 (FoxO3) signaling pathway is involved in the regulation of tumor glucose metabolism reprogramming, malignant biological behavior of cancer cells (proliferative autophagy, apoptosis, inflammatory response, etc.), and in the staging, metastasis, and prognosis of tumors. Additionally, FoxO3 exhibits tumor suppressive effects on GC, which might be a promising therapeutic target in clinic [7]. FoxO3a, a target of AKT and AMPK pathways, is involved in gastric cancer [8].

By analyzing differentially expressed miRNAs in GPL compared to normal gastric tissues, we demonstrated that the expression of miR-194-5p is upregulated in GPL. miR-194-5p regulates glucose metabolism of GPL through its targets FoxO3a. In addition, we also revealed a positive feedback loop between AMPK and AKT.

## 2. Materials and Methods

### 2.1. Animals

Adenocarcinomas of the mouse stomach were induced as previously reported [9]. Briefly, age-matched male specific pathogen-free (SPF) C57/B6 mice were randomly divided into the control group (*n* = 15) and the model group (*n* = 15). The mice were provided with free drinking MNU (N-methyl-N-nitrosourea) solution (120 ppm) (Aladdin Industrial Corporation, Shanghai, China) in light-shielded bottles and were subjected to feed every other day; meanwhile, the control mice were fed normally for 8 weeks. The study protocols were approved by the Ethics Committee of Guangzhou University of Chinese Medicine (No. S2017089). The mice were sacrificed, and the stomach was harvested for further experiments.

### 2.2. Histology, Transmission Electron Microscopy, and Immunohistochemistry

Tissue specimens were embedded in paraffin and stained with hematoxylin and eosin (H&E-stain) and alcian blue (AB-PAS-stain) for mucus characterization. For electron microscopy, the samples were placed in 2% paraformaldehyde and 2.5% glutaraldehyde at 4°C and then stained with 1% uranyl acetate and lead citrate before being observed under the transmission electron microscope. Immunohistochemistry was performed as described by Wei et al. [10] with antibodies to HIF-1*α* (Millipore, MAB5382), vWF (Abcam, ab194405), Ki-67 (Abcam, ab16667), and P53 (Millipore, CBL404).

### 2.3. Western Blot

The following antibodies were used: rabbit FoxO3 antibody (AF6020), rabbit anti-Phospho-FoxO3 antibody (ab154786), rabbit anti-AMPK alpha antibody (ab133448), rabbit anti-Phospho-AMPK alpha antibody (AF3423), rabbit anti-phospho-Akt antibody (CST 4060), rabbit anti-AKT antibody(ab179463), anti-phospho-LKB1 antibody (AF3453), rabbit anti-PCK1 antibody (DF6770), and rabbit anti-GAPDH antibody (AF7021).

### 2.4. Real-time PCR

We adopted the method of 2^-△△^CT to calculate the relative expression levels of miR-194-5p in the gastric mucosa. miR-194-5p was normalized using U6 RNA levels: ^△^CT = CT miR − 194 − 5p − CT U6RNA. The primers for the qRT-PCR were as follows: U6: 5′-CGCTTCGGCAGCACATATACTA-3′ and 5′-CGCTTCACGAATTTGCGTGTCA-3′; miR-194-5p: 5′-CTAGTACCTAGAGGAACCTTTGAAGACTGTTACAGCTCAGCA-3′ and 5′-: AGCTTGCTGAGCTGTAACAGTCTTCAAAGGTTCCTCTAGGTA-3′.

### 2.5. Statistical Analysis

All numerical data are expressed as means ± SEM. Unpaired *t*-test was performed to determine the statistical significance of the difference between two independent groups. *P* < 0.05 was considered statistically significant.

## 3. Results

### 3.1. Pathological Changes of Gastric Mucosa in Precancerous Lesions

In order to explore the influence of MNU on histopathological injury, H&E staining was used to evaluate histopathological alterations. In general, the gastric mucosa of mice in the control group was smooth, moist, and reddish. The gland arrangement of gastric mucosa in the model group was more irregular than that in the control group. In the MNU-treated group, the gastric mucosa was pale, with poor elasticity, and the thickness was fairly uniform. Some of the submucosal vascular hyperplasia, erosion, or granular changes were seen; the epithelial cells were relatively incomplete with uneven cell size and less irregular gland arrangement, which indicated that the GPL model has been successfully established.

**Figure 1:**
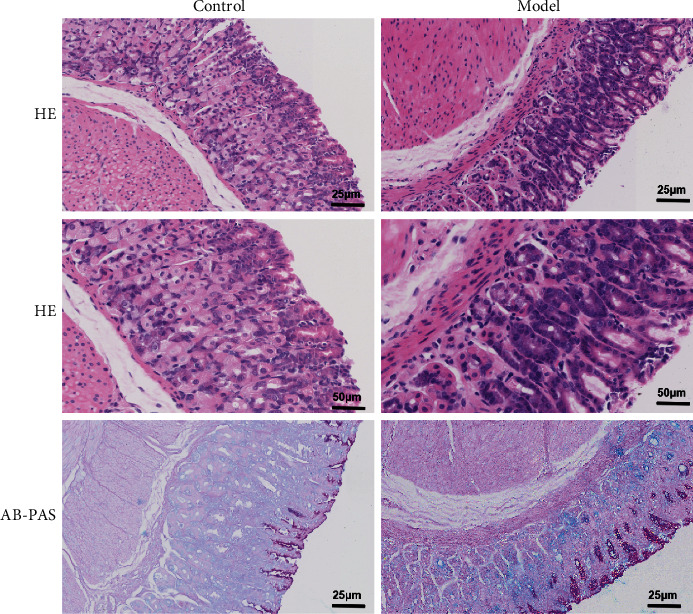
Effect of MNU on gastric mucosal damage in mice. H&E staining (a) and AB-PAS staining (b).

We used the AB-PAS staining to evaluate the degree of IM in gastric tissues [10]. Neutral mucin in the normal mucosa was stained red, while the gastric mucosa in the control group was unstained. After AB staining, the metagenetic tissue of gastric mucosa in mice induced by MNU showed a positive staining. Intestinal metagenetic cells were located in the gastric lumen and lamina propria, and the upper part showed a weak PAS positive staining, with only a small amount of red dye (Figure 1).

### 3.2. Transmission Electron Microscope Analysis

Next, we used transmission electron microscopy to observe the effects of MNU on gastric chief cells and parietal cells and showed that the gastric mucosal gland cavity of the mice in the control group was small and consisted of a single layer of gastric mucosal epithelial cells. The chief cells and parietal cells of control mice were large in size, rich in cytoplasm, and had round or oval nuclei. There were abundant endoplasmic reticulum and pepsinogen particles in the main cells. In parietal cells, abundant mitochondria could be seen.

**Figure 2:**
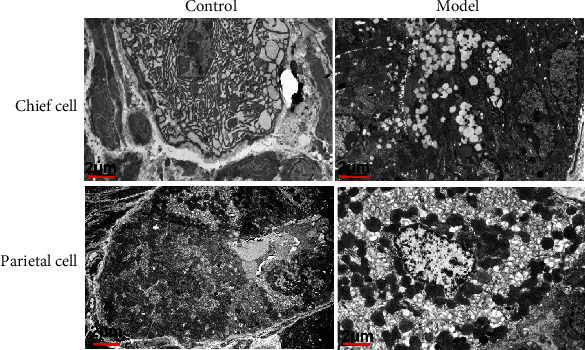
MNU induces ischemia and hypoxia in gastric mucosa. Ultrastructural changes of chief cells (a) and parietal cells (b) in gastric mucosa.

Compared with mice in the control group, the gastric mucosal gland cavity of the model group was significantly expanded; the monolayer became pseudostratified glandular epithelium, with a smaller volume of glandular epithelial cells and with increased electron density. The size of the primary and parietal cells of the mice model group was inconsistent, with less cytoplasm and increased nucleoplasm, and there were a large number of vacuoles of different sizes in the cytoplasm. The chief cell vacuoles were concentrated on the side of the gland cavity and appeared to merge into large vesicles. The remaining main cells formed dysplastic cells with a small volume and a large nucleocytoplasmic ratio. The small vacuoles in the parietal cells were scattered in the cytoplasm, and the original secretory tubule wall membranous structure could be seen in the vacuoles. The fused vacuoles could form sinuses and dysplastic cells. The dense connection between the main parietal cell and the adjacent cell disappeared and transformed into a spindle-shaped epithelial-like transformation cell, indicating ischemia and hypoxia in the gastric mucosa after MNU treatment (Figure 2).

### 3.3. Upregulated Expression of HIF-1*α* and vwf Are Related to the Ischemic Hypoxia Microenvironment of GPL

**Figure 3:**
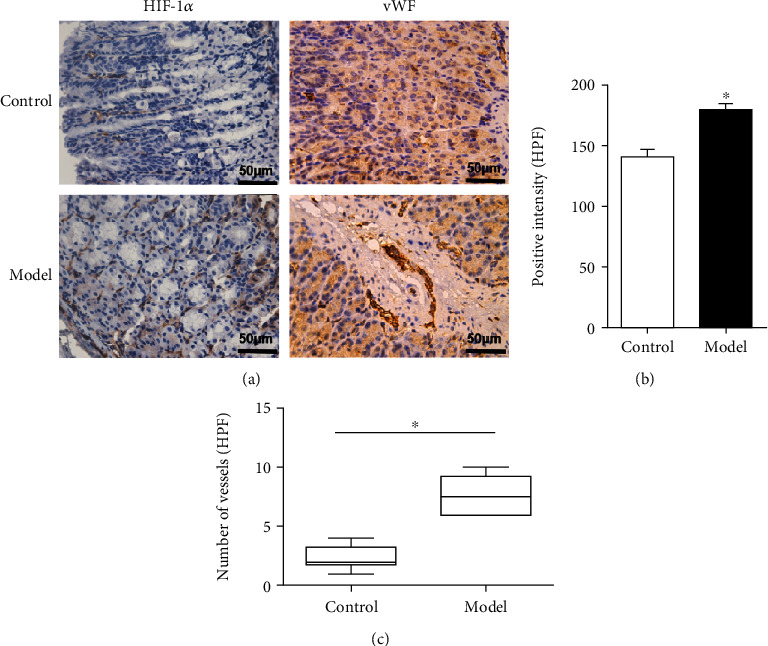
MNU induces a hypoxia transcriptional program in GPL. (a) A tissue array was used to examine the expression of HIF-1*α* and vWF by IHC. The Boxplot diagrams visualizing the number of stained vessels per high power field (HPF). (b) the statistical analysis of HIF-1*α*. (c) The statistical analysis of vWF. ^∗^*P* < 0.01 compared with the control group.

Hypoxia is a typical feature in tumors. In response to hypoxia, HIF-1*α* and von Willebrand factor (vWF) were activated in cells. vWF staining was used to show tiny vessels and individual endothelial cells. To explore the influence of MNU on mucosal hypoxia and ischemia, a tissue array was used to examine expression of HIF-1*α* and vWF by IHC staining. The results revealed that the levels of HIF-1*α* and vWF of MNU-treated mice were both significantly higher than that of control group (*P* < 0.01) (Figure 3).

### 3.4. Upregulated Expression of Ki-67 and P53 Are Related to the Hypoxia Microenvironment of GPL

**Figure 4:**
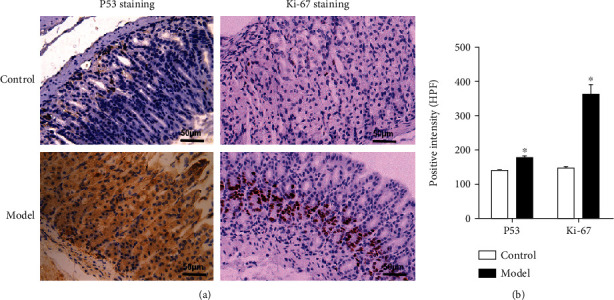
P53 and Ki-67 were increased in GPL. **(**a) IHC staining of P53 and Ki-67. (b) The statistical analysis of P53 and Ki-67. ^∗^*P* < 0.05 compared with the control group.

Ki-67 is a nuclear protein expressed in proliferating cells. P53 mutations can often be detected in the early lesions of gastric cancer [11]. P53 activates hypoxia signaling as early as gastric premalignancy [12]. P53 and Ki-67 have been used as reference indicators for predicting the prognosis of gastric cancer [13, 14].We performed immunohistochemical detection of the P53 and Ki-67 expression in the stomach. Immunohistochemistry showed that the upregulated expression of P53 and Ki-67 are related to the hypoxic microenvironment of GPL compared to the control group (*P* < 0.05) (Figure 4). Altogether, these results suggest that MNU induces gastric mucosal hypoxia in mice.

### 3.5. A novel miRNA-194-5p Is Overexpressed under Hypoxia

**Figure 5:**
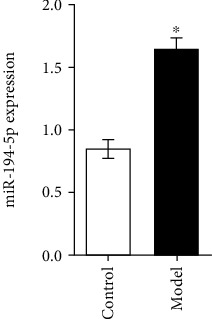
The expression of miR-194-5p increases in the gastric mucosa of GPL. ^∗^*P* < 0.05 compared with the control group.

Studies showed that miR-194-5p provides the basis for the diagnosis and prognosis of gastric cancer, and is closely related to glycolysis [6, 15]. In order to further disclose differentially expressed miRNAs in GPL, qRT-PCR assays were conducted to determine the distinct expression patterns of miR-194-5p in gastric mucosa samples from control mice and mice with GPL. The assays showed that miR-194-5p was significantly increased after MNU treatment (Figure 5). Thus, we demonstrated in this study that upregulation of miR-194-5p in GPL under hypoxic conditions contributed to glycolysis.

### 3.6. Changes in the Protein Expression of Key Genes in FoxO3a Signaling Pathway in the Gastric Mucosa

**Figure 6:**
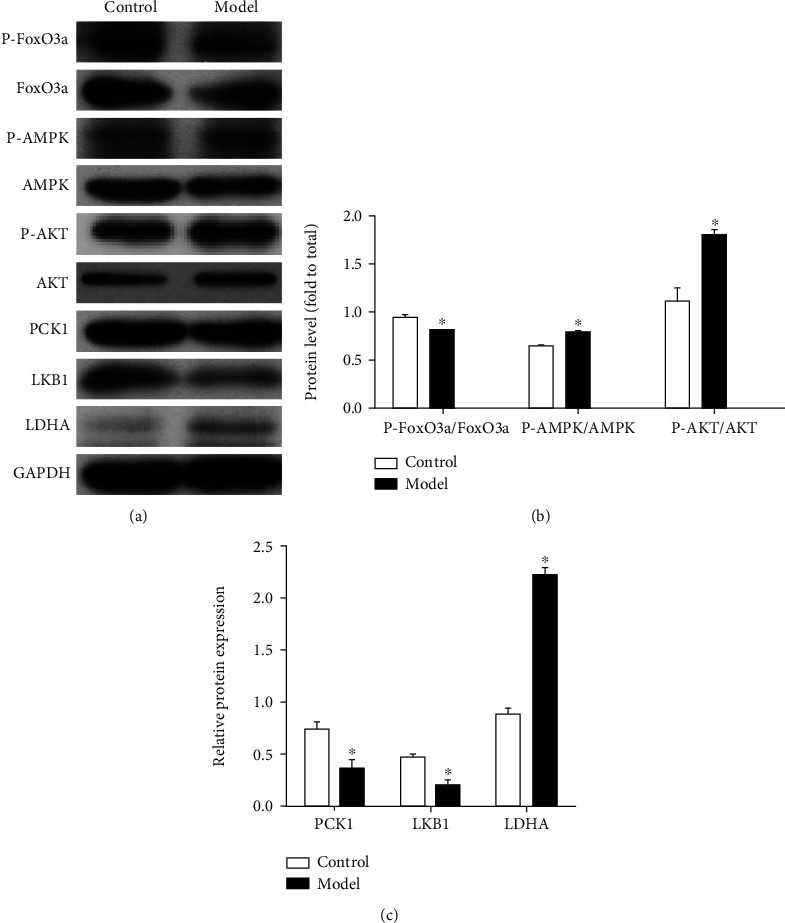
Changes in expression levels of key proteins in the FoxO3a signaling pathway in gastric mucosa. (a) Immunoblot showing protein expression levels of P-FoxO3a, FoxO3a, P-AMPK, AMPK, PCK1, LKB1, and LDHA. (b) The ratio of P-FoxO3a/FoxO3a is lower, and P-AMPK/AMPK and P-AKT/AKT are higher in GPL. (c) The PCK1 and LKB1 decreased, and LDHA overexpressed in GPL, ^∗^*P* < 0.05.

To investigate the effects of MNU on mice, we examined the expression levels of key gene proteins that regulate glycolysis. The ratio of P-FoxO3a/FoxO3a was lower, and p-AMPK/AMPK and p-AKT/AKT ratios were higher in the model group compared with control group. The expression levels of proteins PCK1 and LKB1 decreased, and the expression of LDHA in the gastric mucosa of the model-mice group increased, compared with the control group (Figure 6).

## 4. Discussion

The incidence of gastric cancer is very high, and it has become one of the leading causes of cancer-related deaths worldwide. Changes in GPL are linked to an increased risk of gastric cancer [16]. The reprogramming of cell metabolism is an important sign of cancer and is closely related to the occurrence of tumors. The characteristics of the tumor cells mainly include excessive activation of anaerobic glycolysis and aerobic respiration [17]. Epidemiological studies have shown that elevated blood glucose levels are one of the risk factors for gastric cancer [18]. Therefore, elucidating the factors related to glycolysis is essential for the effective prevention of gastric cancer [19]. This study is consistent with our previous report that there is glycolysis in the gastric mucosa of GPL [20]. MNU induces gastric adenoma and adenocarcinoma due to inflammatory reactions to the alkylating agent [21–23] and has been utilized to induce gastric cancer in mice [9, 24].

In our study, the mouse model showed IM and Dys of gastric mucosa, and microscopic analysis showed that ischemia and hypoxia could be observed in mice receiving MNU. Thus, we proved that the GPL model was successfully established by short-term gavage of MNU in mice.

The formation of tumor is mainly governed by tissue hypoxia, which is a key molecular feature of the tumor microenvironment [25]. In the response to hypoxia, angiogenesis [26], and reprogramming, energy metabolism [27] is involved. Hypoxia-induced HIF-1*α* promotes gastric cancer cell proliferation, invasion, and migration both in vitro and in vivo [28, 29] . The key step in the occurrence and progression of cancer is angiogenesis [30, 31]. vWF, a multimeric plasma glycoprotein, acts as a marker of endothelial dysfunction [32, 33]. More importantly, vWF has been widely used as a biomarker in gastric cancer [34, 35].However, its functional role in GPL is largely unknown. Here, we report that in the gastric mucosa of mice treated with MNU, the expression levels of HIF-1*α* and vWF were significantly increased. These findings demonstrate the causal role of ischemic hypoxia microenvironment-derived vWF in mediating the carcinogenic characteristics of MNU and identify vWF as a new therapeutic target.

In the clinic, the HIF-1*α* expression was correlated with aberrant P53 accumulation and cell proliferation [36]. Ki-67 is a widely used biomarker to estimate the proportion of dividing cells in order to grade tumors [37]. The presence of strong nuclear staining of P53 in the majority of cancer cells is frequently observed [38]. P53 and Ki-67 immunostaining indicated that the mice treated with MNU showed more positive cells compared to the control mice. Altogether, here, we reported that the ischemic hypoxic microenvironment increased the expression levels of HIF-1*α* and vWF, which regulated the level of P53 and promoted the expression of Ki-67.

Hypoxia-inducible miRNAs are engaged in the metabolic reprogramming process. An increasing amount of microRNAs have been found to be related to the carcinogenesis and prognosis of gastric cancer patients [39–41]. miR-194-5p promotes gastric carcinogenesis [42]. Most of the upregulated miRNAs have been linked to gastric cancer. For this study, though, we focused on correlations of miRNAs with GPL. In the present study, we showed that miR-194-5p expression levels increased in the GPL. We suggest that miR-194-5p upregulation is an early event in the cascade of events that lead to the conversion of GPL to cancer, and that it contributes to the establishment of an GPL expression profile through regulation of hypoxic microenvironment.

Transcription of gene-encoding glycolytic enzymes is activated by HIF-1*α*, including stimulation of glycolysis by upregulation of LDHA, which creates an acidic tumor microenvironment [36, 43]. PCK1 is a gluconeogenic enzyme that leads to the regulation of glucose production [44]. In this study, we also observed changes in gluconeogenic enzymes after MNU treatment as the level of LDHA was increased, and the levels of PCK1 decreased in the stomach. In mammalian cells, FoxO3 was involved in glucose metabolism [45, 46] . The regulation of FoxO3a by PI3K-AKT and LKB1-AMPK may play a crucial role in controlling energy balance. The PI3K-AKT pathway controls cell survival, proliferation, and tumor growth, whereas the LKB1-AMPK pathway controls cell cycle arrest and tumor suppression and promotes longevity [47]. FoxO3a, one of the intersections between both the pathways [48, 49], is inhibited by the PI3K-AKT pathway and activated by the LKB1-AMPK pathway [50, 51]. Thus, the PI3K-AKT and the LKB1-AMPK pathways may orchestrate a series of transcriptional (via FoxO3) and posttranscriptional (via mTOR) changes that allow the organism to adapt to changes in the hypoxic status. Our group has characterized the protein expression profiles of PI3K, AKT and mTOR in GPL mice [20, 52, 53]. In the current study, the LKB1 protein expression levels were downregulated, while the ratio of p-AMPK/AMPK and p-AKT/AKT was higher in MNU- receiving mice compared the control mice. Here, we generated the FoxO3a profile of GPL compared to normal mice. We showed evidence here that hypoxia activated miR-194/FoxO3a, inducing glycolytic metabolism reprogramming by upregulating the expression of LDHA and downregulating the expression of PCK1 in GPL.

In summary, our findings identified a novel miR-194-5p, which was upregulated in GPL. Mechanistically, miR-194-5p functioned as an oncogenic miRNA by regulation of the hypoxic microenvironment and subsequently regulated the PI3K-AKT and the LKB1-AMPK pathways via FoxO3a, inducing reprogramming of glycolytic metabolism, and the article is shown in the preprint [54]. More experiments are needed in the future to study the detailed regulatory mechanism.

## Data Availability

The datasets used and analyzed during the current study are available from the corresponding authors on reasonable request.
